# Deciphering Involuntary Movements: A Case of Seronegative Autoimmune Encephalitis With a Kaleidoscopic Presentation

**DOI:** 10.7759/cureus.102647

**Published:** 2026-01-30

**Authors:** Shaheer Arif, Asmeret Demoz, Juan Goyanes, Rozita Khalili, Mahmoud Salhab, Hae Won Shin

**Affiliations:** 1 Department of Neurology, University of Tennessee Health Science Center (UTHSC), Memphis, USA

**Keywords:** adult neurology, autoimmune encephalitis, dyskinesia, epilepsy, hyperkinetic movement disorder, immunotherapy

## Abstract

Seronegative autoimmune encephalitis (SAE) can have a myriad of presenting symptoms including difficult-to-decipher involuntary movements.

A 22-year-old man without significant medical history presented with seizure-like activity, altered consciousness, and numerous extraneous movements. His electroencephalogram (EEG) was difficult to interpret due to excessive myogenic artifact. The patient was intubated due to retained secretions and inability to protect the airway. He was paralyzed, and EEG showed no epileptic activity. He was treated with immunosuppression for SAE after infectious etiologies were ruled out. He clinically improved and was at his cognitive baseline at the last clinic follow-up.

SAE is a rare entity requiring extensive workup and careful exclusion of other etiologies prior to diagnosis. The prognosis varies but can be improved with prompt immunosuppression and appropriate treatment of seizures if present. Involuntary movements need to be carefully evaluated to differentiate movement disorder from epileptic etiology. When titrating medications, diligence is needed so as not to overmedicate, as signified by this case.

## Introduction

Autoimmune encephalitis (AE) is a condition in which the immune system mistakenly attacks the brain, leading to a wide range of neurological and psychiatric symptoms. When no specific causative antibody is identified despite an extensive diagnostic workup, the condition is referred to as seronegative autoimmune encephalitis (SAE) or antibody-negative AE. Some cases of SAE may be explained by novel autoantibodies that have not yet been identified [1].

The estimated incidence of SAE is approximately 0.1 per 100,000 person-years. In adults with SAE, the median age of onset is 38 years, with men comprising 53% of cases. Only 13% of cases occur in individuals over 60 years of age [1,2].

AE is categorized into definite, probable, and possible subtypes. Diagnosis requires a subacute onset of symptoms within three months. A definite diagnosis involves a clinical presentation consistent with a known antibody-associated AE, supportive CSF and/or MRI findings, and identification of an antibody matching the clinical phenotype. A probable diagnosis requires at least two of the following: characteristic MRI findings, inflammatory CSF changes, or brain biopsy showing inflammatory infiltrates. A possible diagnosis is made when one of the following is present: focal CNS findings, new-onset seizures, CSF pleocytosis, or AE-specific MRI abnormalities. In all cases, alternative etiologies must be reasonably excluded [3].

Common clinical features of AE include short-term memory impairment, neuropsychiatric symptoms, sleep disturbances, movement disorders, and new-onset seizures [3].

We present the case of a 22-year-old male patient who presented with a new onset of seizure, multitudes of involuntary movements, and encephalopathy. This case highlights the nuance required for evaluation of involuntary movements and describes the use of paralytics to remove myogenic artifact, when it's obscuring interpretation, from electroencephalogram (EEG) for better evaluation of cortical electrical activity. 

## Case presentation

A 22-year-old man without any significant prior medical, psychiatric, or family history presented to an outside hospital in October 2024 after an episode of seizure-like activity. The patient had altered consciousness, head tremor, right head and gaze deviation, and stiffening of both upper extremities lasting for 2-3 minutes without clonic movements, bowel or bladder incontinence.

In the week preceding this episode, the patient reported feeling unwell with subjective fever, malaise, decreased appetite, nausea, vomiting, and headache. He had no recent travel history but had gone kayaking approximately one month earlier within his city of residence.

His initial physical exam was pertinent for slow mentation and confusion. He was oriented to place, time, and person when given enough time to answer. Motor and sensory exam, reflexes, and coordination on finger-to-nose testing were normal. A continuous head tremor was present that abated when he laid his head down on a pillow.

His condition worsened during admission. He was noted to have eyelid twitching and orofacial dyskinetic activity (Video 1). EEG showed runs of generalized rhythmic delta activity with significant myogenic artifact limiting interpretability. The EEG reader at the outside hospital noted cannot rule out ictal activity. He was sequentially put on anti-seizure medications (ASMs) to suppress the motor activity noted above, including valproic acid (VPA), fosphenytoin (fPHT), levetiracetam (LEV), lacosamide (LCM), and clobazam (CLB). Cerebrospinal fluid (CSF) was obtained, which showed an opening pressure of 18 cm H₂O, and routine studies were as mentioned in Table 1. The CSF meningitis/encephalitis panel was negative. Additionally, the serum autoimmune encephalopathy panel from Mayo Clinic (ENS2) was negative.

**Table 1:** CSF routine studies. CSF: cerebrospinal fluid, WBC: white blood cell.

CSF Routine Studies	Test Value	Reference Value
WBC	7/mm^3^	0-5/mm^3^
Protein	52.5 mg/dl	15-40 mg/dl
Glucose	64 mg/dl	45-75 mg/dl

**Video 1:** Dyskinetic facial movements during index admission.

**Figure 1:**
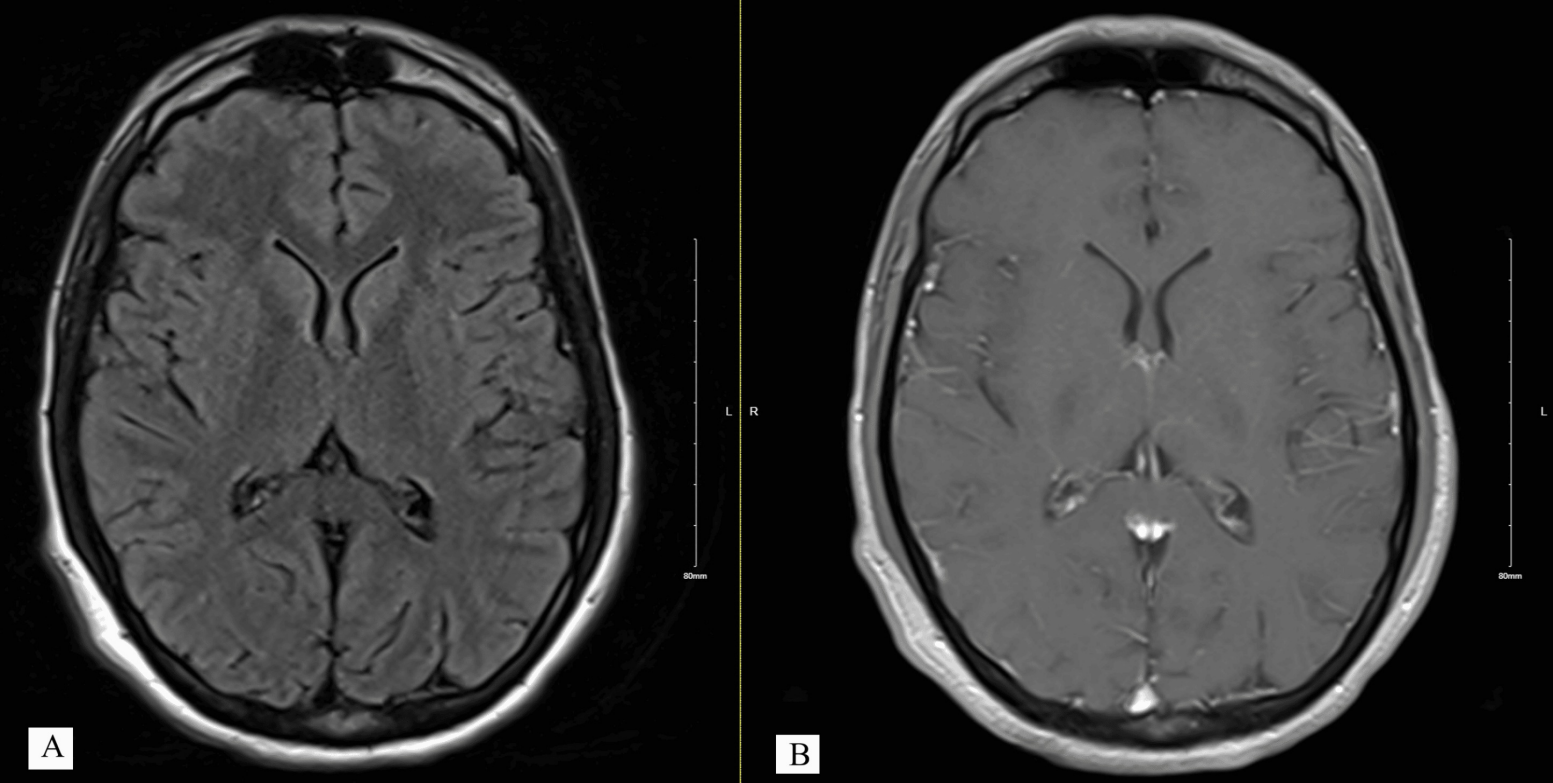
Initial MRI brain within normal limit. A. T2 FLAIR MRI brain on the left, B. T1 Post contrast MRI brain without abnormal contrast enhancement FLAIR: Fluid-attenuated inversion recovery.

He was kept on antimicrobial coverage and worked up for tick-borne diseases, which were negative. Urine drug screen was negative. He had clinical worsening manifested by visual hallucinations, dysphagia, cramps, memory impairment, diplopia, and ataxia. Brain MRI with and without contrast appeared normal initially (Figure 1), and two repeat MRIs at 10 days and 21 days from the initial scan also showed no abnormalities. He was treated with 1 gram IV methylprednisolone for three days and underwent three sessions of plasmapheresis (PLEX 3 sessions) for suspected AE. No clinical improvement was evident, prompting transfer to our hospital for neuro intensive care unit (NICU) level of care and continuous video EEG (cvEEG) monitoring.

On arrival at our hospital, the patient exhibited facial twitching and bilateral upper extremity dyskinetic movements, which were more pronounced on the left than the right. He was not protecting his airway and was subsequently intubated, during which large amounts of retained secretions were visualized. He was placed on IV midazolam and ketamine drips for sedation and seizure suppression along with dexmedetomidine. His ASMs were adjusted to brivaracetam (BRV), LCM, VPA, and CLB.

**Figure 2:**
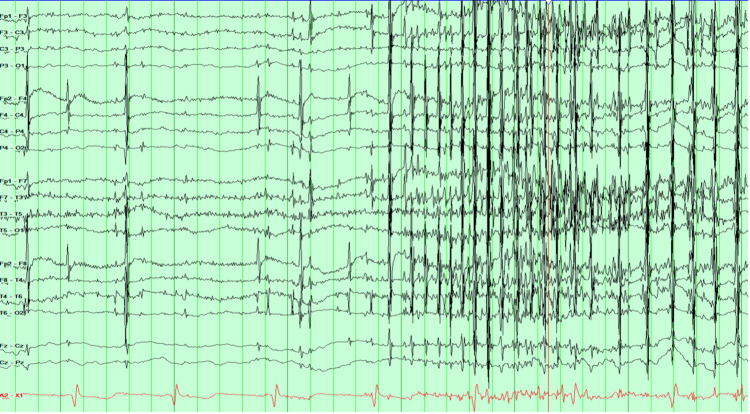
EEG in double banana montage showing EMG artifact. EEG: Electroencephalogram, EMG: Electromyographic

EEG was reported as potential diffuse runs of 3-7 Hz generalized spike-and-wave and polyspike-and-wave discharges with a myogenic artifact making interpretation difficult (Figure 2). The patient continued to experience stimulus-induced stereotyped episodes with facial and predominant left arm jerking. Due to persistent concerns for super-refractory status epilepticus, the number of PLEX sessions was increased to 10. He was started on methylprednisolone taper starting at 60 mg twice a day. A new CSF sample was obtained; the autoimmune encephalitis Mayo Clinic CSF panel (ENC 2) was sent and returned negative. Extensive infectious workup on CSF, including viral infections, and atypical organisms, including arbovirus battery, was negative. Paraneoplastic screening, including testicular ultrasound, was also negative.

**Figure 3:**
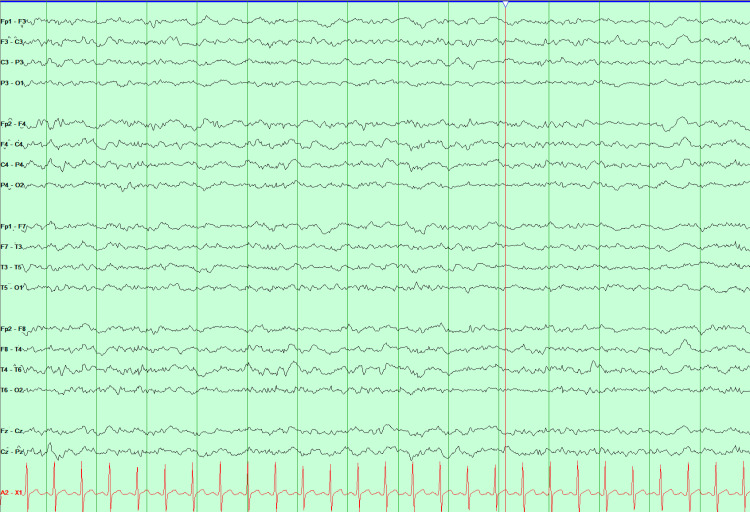
EEG in double banana montage showing diffuse slowing after muscle paralysis. EEG: Electroencephalogram

The patient was paralyzed to assess underlying cerebral activity without myogenic artifacts; no discharges were observed (Figure 3). The earlier EEGs were compared, and it was determined that potential discharges described in the previous reports were misinterpreted due to a myogenic artifact that was unusually high amplitude, and in instances stimulus induced. The patient was gradually weaned off ketamine and midazolam drips. After completion of PLEX, he was given rituximab 1000 mg with plans to repeat the dose after two weeks. A three-day course of anakinra was given on discretion of the NICU physician. Subsequently, facial and upper extremity myoclonic movements reoccurred, but no epileptiform activity was seen on cvEEG.

Electromyography and nerve conduction studies (EMG/NCS) ruled out neuromyotonia. Since the patient was following commands, no further ASM escalation was done. His final ASM regimen was LEV (switched from BRV due to cost), LCS, CLN, and perampanel (PER). He was discharged to inpatient rehabilitation with tracheostomy and percutaneous endoscopic gastrostomy tube after receiving two doses of rituximab 1000 mg.

At discharge, the patient was awake, oriented to time, place, and person, but severely dysarthric. No cranial nerve deficits were noted. Motor exam was remarkable for continuous spontaneous dyskinetic movements involving the orofacial region and upper extremities. There was bilateral arm weakness with left arm spasticity at the elbow, hyperreflexia, clonus in the feet, and left foot drop.

The patient was discharged from rehabilitation after two weeks in December 2024. He was admitted to another tertiary care hospital for ASM adjustment and reduction of polypharmacy. He was placed on cvEEG and diagnosed with epilepsia partialis continua due to left thumb clonic twitching, with similar EEG findings previously reported in our center. Final ASMs on discharge were CLB 20 mg daily, LEV 1000 mg twice daily, LCM 300 mg twice daily, and PER 8 mg nightly. Other medications such as bromocriptine, amantadine, ropinirole, and baclofen that were used for tremors and spasticity were gradually discontinued to reduce polypharmacy, resulting in further improvement in tremors. A repeat CSF analysis during this admission was unremarkable; autoimmune epilepsy panel from the Mayo Clinic (EPC2), IgG index, and oligoclonal bands were all negative. The patient received his third and fourth doses of rituximab, each at 375 mg/m². MR spectroscopy showed a normal brain but relatively reduced N-acetyl-aspartate to creatine ratio in the medial temporal lobes, left greater than right; a finding that was considered non-specific. He was discharged home in stable condition.

During his most recent epilepsy clinic visit, he showed significant improvement and had returned to his cognitive baseline. However, he still exhibited head tremor with dyskinesias (Video 2), frequent eye blinking, left hand postural tremor, and left thumb twitching. It was decided to taper off PER and continue LEV 1000 mg twice daily, LCM 150 mg twice daily, and CLB clobazam 20 mg nightly.

**Video 2:** Patient with residual orofacial dyskinesia.

## Discussion

SAE is a rare entity. Due to its rarity, most clinicians lack experience with its clinical course and optimal management strategies. In our literature search, most descriptions of SAE involved children under 18 years and adults older than 60 years of age [4-7]. Our case adds to the literature by providing a detailed account of the timeline and progression of the clinical course in a young adult patient diagnosed with SAE.

Lee et al. developed a clinical outcome scale, ‘RAPID’, to help clinicians predict prognosis in patients with SAE [7]. The score assigns one point each to refractory status epilepticus, age greater than or equal to 60, antibody-negative probable AE, infra-tentorium involvement on brain MRI, and delay of immunotherapy greater than or equal to one month. A RAPID score cut-off of 2 is associated with poor two-year outcomes (sensitivity 81.3%, specificity 66.3%) [7]. Our patient had a RAPID score of 0. The favorable outcome observed was consistent with the prediction of the score, although the score did not account for possible AE, as in our case. The absence of abnormalities on brain MRI likely correlates with a better prognosis, based on the assumption that extensive brain parenchymal damage has not occurred to produce detectable changes on imaging.

Since AE is a rare entity, no randomized trials have investigated the efficacy of immunotherapy (IT) in its treatment. However, numerous retrospective studies suggest that IT can improve outcomes [7-9]. Escalation of IT is typically conceptualized as a tiered approach. The first tier includes intravenous steroids, intravenous immunoglobulin, and plasma exchange (PLEX). The second tier, used in cases unresponsive to initial therapy or in severe cases, consists of rituximab or cyclophosphamide [9,10]. Whether immunotherapy is continued after the acute phase depends on residual disease, usually assessed based on clinical response and biomarkers such as repeat MRI and CSF findings.

Our patient was treated with high-dose steroids, started early on rituximab, and received a trial of three doses of anakinra. Cyclophosphamide was considered but not administered due to clinical improvement.

Our case highlights another aspect of AE presentation that can make management tenuous: the presence of involuntary movements, including tremors, myoclonus, clonus, and dyskinetic movements [11]. These can sometimes be confused with one another. Myoclonic and clonic movements are often associated with electrographic seizures and should be treated promptly to prevent worsening and progression to status epilepticus [12,13]. The other types of movements are generally managed symptomatically if they are bothersome to the patient [14].

The dilemma arises when it is unclear which movement type is present, especially when the patient is unresponsive, and whether to escalate ASMs [15]. In our case, paralytics were used to assess underlying cerebral activity by removing muscle artifact from EEG recordings, as excessive myogenic activity can obscure cerebral signals. This underscores the importance of caution in labeling extraneous movements as epileptic.

Initially, our patient's tremors and dyskinetic movements were managed with spasmolytics and dopaminergic medications; however, these were gradually withdrawn due to lack of apparent benefit. Clinicians should exercise caution when adding medications solely to suppress extraneous movements without clear clinical benefit.

## Conclusions

SAE can be a diagnostic challenge, requiring a high index of suspicion. Early initiation of treatment is crucial for better outcomes. A careful assessment of extraneous movements' semiology, along with correlation with EEG, is essential to distinguish epileptic from non-epileptic movements and to avoid over-treatment.
